# Factor structure of school readiness skills: conceptual vs. statistical distinctions

**DOI:** 10.3389/fpsyg.2023.962651

**Published:** 2023-07-10

**Authors:** Robert J. Duncan, Kirsten L. Anderson, Jennifer K. Finders, David J. Purpura, Sara A. Schmitt

**Affiliations:** Department of Human Development and Family Studies, Purdue University, West Lafayette, IN, United States

**Keywords:** early childhood education, school readiness, executive function, achievement, latent factor analysis

## Abstract

**Introduction:**

School readiness skills are a broad set of abilities that children develop in early childhood that support achievement once they enter formal schooling. Three components of school readiness skills are of focus in the current study: executive function (EF), language/literacy, and mathematics. The current study examines to what extent 13 direct assessments of these skills statistically align with theoretical models for distinct construct- and timepoint-specific latent factors.

**Methods:**

The sample included 684 children (52.34% male; 42% Black/African American; *M*_age_ = 4.80 years in the fall of prekindergarten) assessed in the fall and spring of the prekindergarten year.

**Results:**

Factor analyses revealed the most statistical support for a model with a latent random intercept across timepoints and constructs, along with timepoint-specific latent factors in the fall and spring of prekindergarten (independent of the random intercept). The timepoint-specific latent factors primarily consisted of early literacy and mathematics assessments.

**Discussion:**

These findings challenge commonly held practices of creating construct-specific latent factors in early childhood research and, to a lesser extent, timepoint-specific latent factors without consideration of the substantial shared variance across different constructs and timepoints. Implications for the factor structure and developmental theory of school readiness skills are considered, as well as practical considerations for future research.

## 1. Introduction

School readiness reflects a broad set of skills that children develop in the early childhood years which support success in formal school environments and subsequent achievement (Ackerman and Barnett, 2005; Snow, 2006; Duncan et al., 2007). These interrelated skills span across a number of conceptual domains, including cognition and emergent achievement (e.g., mathematics, literacy; Duncan et al., 2007), behavior (e.g., self-regulation; Blair and Raver, 2015), physical development (e.g., fine motor skills; Cameron et al., 2012), and socio-emotional development (e.g., emotional regulation; Graziano et al., 2007). The current study focuses on three constructs primarily within the cognitive domain of school readiness that have received considerable attention due to their relatively strong associations with future academic achievement: executive function (EF), language/literacy, and mathematics (e.g., Duncan et al., 2007, 2020).

Given their multidimensionality, these constructs (i.e., EF, language/literacy, and mathematics) are often evaluated using a number of different tasks in early childhood. In cases where multiple tasks are used to capture a single construct of interest (e.g., EF), prior work has often relied upon latent factor analyses. That is, the latent factor captures shared variance among observed (or indicator) variables of the construct, and thus does not include task-specific measurement error (though other types of error may remain, e.g., due to missing indicators of the underlying skill of interest). Recent work, however, has raised concerns about the models most commonly used when examining school readiness skills due to the potential for substantial bias, even if these models are well-fitting (Camerota et al., 2020; Rhemtulla et al., 2020). The primary assumption with the latent factor approach is that what is shared variance across tasks of a common construct would be the intended construct (e.g., EF; Nesbitt et al., 2019), and not confounding factors that influence performance across tasks of that construct (e.g., language abilities, motivation, general cognitive skills).

Therefore, the current study is interested in understanding the potential bias when modeling school readiness skills (i.e., EF, language/literacy, and mathematics) using latent variable analyses. We test if the shared variance of specific constructs (e.g., EF) is statistically distinct from the shared variance of other constructs (e.g., mathematics or language/literacy). Using EF as an example, if the latent construct is statistically distinct, it would be reasonable to assume that the shared variance of EF tasks represents EF and not the other constructs of mathematics or language/literacy. However, if the shared variance of EF tasks and tasks of the other constructs are not statistically distinct, then it would be reasonable to assume that the shared variance of EF tasks represents EF and the other skills that influence performance across constructs. In the latter scenario where the data does not support the assumption that the latent constructs are statistically distinct, then any cross-domain associations that are tested in these models would be biased due to the common shared contributors to the latent factors. For example, language or motivation (i.e., confounding factors) may be what is shared across latent factors of EF and mathematics more so than the intended underlying construct of interest. Moreover, EF skills may be captured in the latent factor of mathematics.

Similarly, prior studies have highlighted the role of relative between-child stability that exists in skills across timepoints and its potential to upwardly bias estimates in cross-lagged and autoregressive paths (Hamaker et al., 2015; Berry and Willoughby, 2017). This issue arises in autoregressive or cross-lagged associations when relative between-child stability exists within (or across) constructs. The primary concern is that other factors, such as stable environmental influences, but not purely the prior ability level, contribute to children’s skills over time. Based on prior studies, we consider that it is likely a combination of both prior ability levels and relatively stable between-child factors that contribute to children’s performance over time on these skills (Bailey et al., 2014; Nesbitt et al., 2019). Thus, the primary concern with regard to bias in longitudinal models is that if relative between-child stability factors are not accounted for in the statistical approach, those factors could upwardly bias autoregressive and cross-lagged effects in the analyses (Hamaker et al., 2015; Berry and Willoughby, 2017).

Fortunately, the potential for both types of biases in analyses can be examined by testing multiple theoretical latent variable models to understand the extent to which the data is consistent with construct- and/or timepoint-specific latent factors vs. the shared, stable variance across construct and timepoints. Specifically, nine *a priori* latent variable models are tested to understand the consistency of the observed data with distinct theoretical (and statistical) models that focus on shared variance across constructs and timepoints vs. construct- and timepoint-specific variation. The primary research objective we are informing is to what extent latent variables of specific constructs and timepoints are capturing shared variation that is construct- and/or timepoint-general (i.e., variation not unique to the construct or timepoint).

### 1.1. Measurement of school readiness skills in prekindergartners

The cognitive domain of school readiness has received considerable attention in the field of early childhood development and education. The constructs of EF, language/literacy, and mathematics are often found to be robust predictors of concurrent cross-domain skills and future academic achievement (Duncan et al., 2007; McClelland et al., 2014; Schmitt et al., 2017; Duncan et al., 2020). Importantly, these skills are malleable to environmental influences, including prekindergarten curricula and interventions; indeed, previous studies have suggested that there are a number of early interventions associated with improved skills in these domains (e.g., Clements and Sarama, 2007; Lonigan et al., 2013; Weiland and Yoshikawa, 2013; Schmitt et al., 2015). However, it is still unknown how to best model these constructs statistically in observational longitudinal studies to inform theory, especially when multiple tasks of a given construct exist. Importantly, it is necessary to decipher to what extent associations are unique to a specific construct and developmental period or they are shared more generally across constructs and timepoints for understanding cross-domain and developmental associations.

Prior studies have not fully informed *how* measures of EF, language/literacy, and mathematics should be modeled statistically when multiple tasks are assessed within each construct over time (i.e., the role of shared and unique variance across constructs and timepoints). The current study aims to address this existing gap in the literature by examining statistical support for distinct theoretical models when multiple assessments of each construct exist across the prekindergarten year. Within EF research, the vast number of measures that exist in the field to assess children’s abilities has been referred to as “measurement mayhem” (Morrison and Grammer, 2016), though it is also common for researchers to utilize more than one measurement tool when evaluating early mathematics and language/literacy skills (Weiland and Yoshikawa, 2013; Cabell et al., 2015; Purpura et al., 2017a; Justice et al., 2018). Given that many assessment instruments exist for each of these three constructs, researchers may feel compelled to collect multiple assessments of each construct and create latent factors to capitalize on shared variance of tasks within the same construct and try to eliminate task-specific measurement error. However, this method of latent variable modeling may introduce bias, such as other confounding factors that contribute to performance across tasks that are not construct-specific (e.g., a mathematics assessment requiring language abilities, motivation, and/or EF skills; Rhemtulla et al., 2020). We argue these presumed latent variable models should be compared to alternative plausible theoretical models to examine the consistency of the data with different specifications (e.g., Bailey et al., 2018; Camerota et al., 2020).

It is important to note that the current study does not try to inform which construct is most important for future achievement or school readiness. Rather, the current study addresses a more fundamental research question and current gap in the field: does the statistical evidence align with the conceptual distinctions for constructs and timepoints when modeling these skills using a latent variable approach? If statistical distinctions do not align with conceptual distinctions, then statistical models that employ latent variables based only on conceptual justifications, and not statistical, are likely subject to bias. Further, any cross-domain and cross-time statistical inferences drawn from these models are likely biased. Based on prior evidence (e.g., Berry and Willoughby, 2017; Nguyen et al., 2019), there is reason to hypothesize statistical distinctions will not be fully consistent with the commonly held conceptual distinctions of unique construct-specific and timepoint-specific latent factors. However, we do not assume there will necessarily be dichotomies regarding the variation at the construct- or timepoint-level (i.e., one or the other), rather there could be a combination of both shared and unique variation. Finally, we did not consider higher order factors in our analyses, only the extent to which construct- and timepoints-specific captured unique and shared variation.

### 1.2. Statistical and conceptual considerations of latent factors

The focus of the current study is on the degree to which there are statistical distinctions among EF, language/literacy, and mathematics during the fall and spring of the prekindergarten year when using latent variable modeling. Therefore, emphasis is placed on the statistical support for multiple potential models, which is important to consider in light of recent criticism for bias in misspecified latent variable approaches and the implications for interpretations (Camerota et al., 2020; Rhemtulla et al., 2020). The emphasis of the current study is to inform which models are most consistent with the observed data using nine *a priori* model specifications for the factor structure of cognitive school readiness skills.

#### 1.2.1. Construct-specific vs. construct-general factor structure

The first primary question is the degree to which there are statistical distinctions of construct-specific vs. construct-general factors for cognitive school readiness skills. Specifically, this addresses to what extent EF, language/literacy, and mathematics assessments (i.e., observed variables) load onto unique, construct-specific latent factors, a common, shared latent factor, or some combination of both. Models that include construct-specific latent factors for EF, language/literacy, and mathematics assume a theoretical structure of the data where tasks of one construct share meaningful variation with other tasks within the same construct that is distinct from tasks of other constructs. That is, EF tasks share variation with other EF tasks that is unique from the variation that would be shared among mathematics and language/literacy tasks. Related, but distinct, the prior EF literature in early childhood has typically focused on the extent to which the three components of EF (inhibitory control, cognitive flexibility or attention shifting, and working memory) load onto a single latent factor or onto distinct, component-specific latent factors (Wiebe et al., 2008). Generally, findings have been consistent with a single underlying EF factor, and this has been the assumed theoretical perspective when a single EF latent variable is used to predict mathematics and/or literacy performance (Schmitt et al., 2017; Nesbitt et al., 2019). However, it is unclear if the statistical support for a single EF factor is influenced by shared variance across other constructs as well. That is, to what extent is data consistent with an underlying EF latent factor being statistically distinct from an underlying mathematics or language/literacy latent factor?

Of the nine *a priori* models we tested, five models assume that shared variance is not at the construct-specific level but is attributable to other factors that influence performance across constructs (Supplementary Figures 1–4, 7). The additional constraints on these models (random intercept or freely estimated loadings) either restrict tasks to relate equally to other tasks (i.e., the random intercept; Models 2, 4, and 7) or allow some tasks to relate more strongly to other tasks by not forcing it to be construct-specific (i.e., freely estimated loadings; Models 1 and 3). In both types of models, tasks are not assumed to relate more closely to tasks that tap the same construct compared to a different construct. However, because random intercepts have unstandardized loadings constrained equal to 1, the additional restriction is made that regardless of the intended construct, all tasks relate similarly in magnitude. Two models (Supplementary Figures 5, 6) assume that shared variation is only at the construct-specific level (i.e., unique to each of the three domains: EF, language/literacy, and mathematics). The distinction between these models is whether this construct-specific variation is either timepoint-general (Model 5) or timepoint-specific (Model 6). Two models (Supplementary Figures 8, 9) assume it is a combination both construct-specific and construct-general variation.

One argument against construct-specific factors is that there is limited evidence for a statistical distinction in prior work. For instance, a recent study examining the association between EF and mathematics in early childhood found that (1) EF tasks are generally more highly correlated with mathematics tasks than they are with other EF tasks, and (2) mathematics tasks typically loaded more strongly onto a “latent EF” factor than did EF tasks (Nguyen et al., 2019). This study suggests that an underlying latent EF factor may not be distinct from mathematics abilities. This is problematic from an inference standpoint if they are modeled as distinct latent factors because the shared variance across the EF and mathematics tasks may be attributable to things other than those specific constructs (e.g., language, motivation). However, this study did not examine the additional construct of language/literacy, which has also been found to correlate closely with early mathematics (Purpura et al., 2017a) and, to a lesser extent, EF (Fuhs et al., 2014; Schmitt et al., 2017).

One explanation for why EF tasks could relate similarly to mathematics tasks as to other EF tasks is “task impurity.” For example, task impurity is when EF tasks that are intended to capture inhibitory control may rely on another component of EF as well (e.g., cognitive flexibility; Miyake et al., 2000; Tarle et al., 2019). However, task impurity may not be specific to EF tasks in early childhood, as all school readiness assessments likely rely on a combination of language abilities and domain-general cognitive skills (e.g., motivation, engagement, attention, persistence). For example, a mathematics task that requires children to compare quantities may rely on domain-specific skills as well as EF skills, such as working memory (Purpura et al., 2017b). If the statistical evidence only supports a common latent factor that shares variance across EF, language/literacy, and mathematics tasks, then models that only estimate construct-specific latent factors will be biased due to the factors that contribute similarly to children’s performance across constructs. Acknowledging this may not be a pure dichotomy, we model the potential for construct-specific variation, construct-general variation, or both in our model specifications.

#### 1.2.2. Timepoint-specific vs. timepoint-general factor structure

The second primary question is the degree to which there are statistical distinctions between timepoint-specific latent factors vs. a timepoint-general factor structure of cognitive school readiness skills. Specifically, the models address to what extent assessments at fall of prekindergarten and spring of prekindergarten load onto timepoint-specific latent factors, a common, shared latent factor (i.e., relative between-child stability throughout the year), or a combination of both. This is important because of the potential for relative between-child stability to create upwardly biased estimates in cross-lagged panel models (Berry and Willoughby, 2017). Most models of developmental processes have typically assumed that the fall and spring of prekindergarten capture meaningful unique variation only. Children are in the process of developing these skills, and many prekindergarten programs are now focused on boosting school readiness skills or their antecedents (e.g., resisting impulses, basic counting, alphabet knowledge, letter sounds). Therefore, children’s performance on these assessments are changing (often rapidly) over the prekindergarten years and variation in environmental experiences are assumed to lead to meaningful variation in how these skills change over the year. This conceptual framework aligns with the perspective of statistical models that use autoregressive or cross-lagged effects of skills at prior timepoints to predict skills at subsequent timepoints and to seek factors that relate to the residual change in skills.

Much like the conceptual distinctions, it would seem intuitive that there would be unique timepoint-specific latent factors (i.e., variation that is not stable across time). The fall and spring of prekindergarten represent distinct periods of development, and although skills across these timepoints are typically closely related, it seems unlikely that there would only be meaningful variation in relative between-child stability across the prekindergarten year. However, recent studies on longitudinal methods challenge prevailing perspectives on how skill development within children should be modeled (Hamaker et al., 2015; Berry and Willoughby, 2017). Namely, random-intercept cross-lagged panel models have called attention to relative between-child stability in skill performance across timepoints. Typically, these types of models yield estimates that reflect both relative between-child stability in skills, as well as autoregressive and cross-lagged effects (Bailey et al., 2014; Nesbitt et al., 2019). This would be consistent with a combination of unique timepoint-specific variation and relatively stable variation across time.

Of the nine *a priori* models we test, four assume that shared variance is not at the timepoint-specific level but is due purely to relative between-child stability in skills (Supplementary Figures 1, 2, 5, 8). Models 1 and 2 test these as either single latent factors or random intercepts regardless of construct-specific performance, and Models 5 and 8 test these assumptions while differentiating for the constructs. Thus, the primary distinction would be that random intercepts force all tasks to relate similarly in magnitude to underlying factor (e.g., around 0.40). Conversely, the single latent factor with freed factor loadings would allow some tasks to relate more strongly than others and not impose any assumptions about which tasks at which timepoints relate more closely to the underlying factor. Three models (Supplementary Figures 3, 4, 6) assume that shared variation is only at the timepoint-specific level and not due to relative between-child stability. In other words, no latent factor is specified across the two timepoints, rather there are timepoint-specific latent factors (Model 3), timepoint-specific random intercepts (Model 4), or construct and timepoint-specific latent factors (Model 6). Two models (Supplementary Figures 7, 9) assume it is a combination of both timepoint-specific and relative between-child stability. It is important to note that in all *a priori* models we included cross-timepoint residual correlations among specific tasks. This was done because correlations within a specific task across time are typically more highly related (compared to other tasks at a given timepoint). This could be due to the measurement characteristics of specific tasks or that children may simply do better or worse on a specific task for other reasons. Thus, one type of cross-timepoint shared variation is always modeled with these residual correlations.

### 1.3. The current study

The research objective in this study was to examine the statistical support for theoretical factor structure models for 13 direct assessments, including EF, mathematics, and language/literacy, assessed in the fall and spring of children’s prekindergarten year (i.e., approximately 6 months a part). There are two hypotheses based on prior research: (1) data will not support that the latent constructs are statistically distinct based on findings that mathematics tasks consistently load more strongly onto latent EF than EF tasks (Nguyen et al., 2019) and other work showing cross-domain correlations for these three constructs similar in magnitude to within-domain correlations (e.g., Purpura et al., 2017b); and (2) substantial variability will be shared across timepoints given relative between-child stability observed on these dimensions during this developmental period, though there will likely remain some meaningful timepoint-specific variation as well (Bailey et al., 2014; Nesbitt et al., 2019). If the data are not statistically consistent with conceptual distinctions (i.e., they capture primarily cross-construct or cross-timepoint variation), then research studies should reevaluate the use of only preconceived latent variable models based on conceptual justifications and consider alternative theoretical specifications (or other analytic strategies). Additionally, multiple latent variable models should be estimated to better inform theory, otherwise incorrect conclusions may be drawn from unchallenged assumptions (Camerota et al., 2020; Rhemtulla et al., 2020).

## 2. Materials and methods

### 2.1. Participants

The data for this study were collected in the United States as part of a Midwest state-wide initiative assessing school readiness skills among children receiving childcare subsidies to attend prekindergarten programs that differed based on the Quality Rating and Improvement System level (a state-level quality metric based on child care characteristics). This quasi-experimental design compared school readiness skill development of children enrolled in high-quality prekindergarten programs (treatment) to children of similar demographic backgrounds who were enrolled in lower-quality programs (comparison). Data collection took place throughout children’s prekindergarten school year and included 3 cohorts of children (2015–2019). Recruitment strategies varied across cohorts and between the treatment and comparison groups to address the specific aims of the larger evaluation study. In general, however, recruitment was a multistep process. First, members of the research team or the state agency that oversees early childcare and education identified prekindergarten programs that met eligibility criteria for the study. In most cases, the number of eligible programs exceeded our assessment capacity; thus, the research team selected programs randomly or based on the specific aims of the study. After individual providers consented to participate in the study, children eligible to participate in this study were recruited; those whose parents provided informed consent were enrolled in the study.

Of our final sample of 684 students (52.34% male; *M*_age_ = 4.80 years), 54.24% were enrolled in Cohort 1, 27.34% were enrolled in Cohort 2, and 18.42% were enrolled in Cohort 3. The majority of children (66.67%) were enrolled as part of the treatment group, while 33.33% were in the comparison group. Reflecting the demographic makeup of the counties assessed, 42.07% of children were Black/African American, 31.12% of children were White, 13.11% were Hispanic or Latinx, and 10.37% of children were multiracial. The average household income of participants was $18,481.45, and all children in the sample were from households with income at or below 127% of the federal poverty level.

### 2.2. Procedures

This study was approved by the institutional review board (IRB) at [removed for blinding]. The research assistants who administered the assessments in this study were trained and certified by research team staff. Data collection procedures for the comparison and treatment groups were identical. After receiving the child’s assent, research assistants administered the assessments to children individually at their school (e.g., in a quiet corner of the classroom; in the hallway) during the fall and spring semesters of prekindergarten. The assessments were administered in either a single session of approximately 60–90 min or spread across multiple shorter sessions of 20–30 min each. The child was permitted to take breaks when necessary. All children received stickers as incentives, regardless of whether they completed the assessments.

### 2.3. Measures

A total of 13 assessments were administered to children in this study: three assessments of executive function (EF), four assessments of language/literacy, and six mathematics assessments. In the following paragraphs, we briefly describe each of the assessments and their psychometric properties; for a detailed description of their respective administration protocols, please see Supplementary Table 1.

#### 2.3.1. EF

In total, this study included three measures of EF; these measures assess children’s inhibitory control, working memory, and/or cognitive flexibility, which are commonly accepted as the three core components of EF (Diamond, 2013). The first EF measure, the Head-Toes-Knees-Shoulders task (McClelland et al., 2014), is a direct assessment of working memory, inhibitory control, and cognitive flexibility and is designed for children between the ages of 3 and 7. The second assessment of EF skills was the Day/Night Stroop task (Gerstadt et al., 1994), which assesses children’s ability to inhibit automatic responses (i.e., inhibitory control). The third EF task, the Dimensional Change Card Sort (Zelazo, 2006), was only administered to children in Cohort 3 and is an assessment of cognitive flexibility (Zelazo, 2006).

#### 2.3.2. Language/literacy skills

We measured several components of children’s early language/literacy skills, including vocabulary skills, letter and word knowledge, and emerging reading skills. The fourth edition of Peabody Picture Vocabulary Test (Dunn and Dunn, 2007) was used to assess receptive vocabulary skills. Letter and word knowledge were measured using the Letter-Word Identification subtest of the Woodcock-Johnson Test of Achievement IV (Schrank et al., 2014) and the Letters subtest of the Bracken Basic Concept Scale: Third Edition-Receptive (Bracken, 2006). Finally, emerging reading skills (e.g., print knowledge, phonological awareness) were assessed using the Get Ready to Read Revised (Lonigan and Wilson, 2008).

#### 2.3.3. Early mathematics skills

To measure a wide range of early mathematics skills, six different early mathematics skill assessments were administered. To assesses basic early mathematics skills (e.g., counting, addition, and subtraction) we used the Applied Problems subtest of the Woodcock-Johnson Test of Achievement IV (Schrank et al., 2014), a brief version of the Preschool Early Numeracy Skills Screener (Purpura et al., 2015; Purpura and Lonigan, 2015), and the Numbers/Counting subtest of the Bracken Basic Concept Scale: Third Edition-Receptive (Bracken, 2006). In addition to these measures of broad early mathematics skills, we also assessed more specific aspects of early mathematics skills, including mathematics language, cardinality, and numeral identification. Mathematics language was assessed using the Preschool Assessment of the Language of Mathematics (Purpura and Logan, 2015; Purpura and Reid, 2016), cardinality was evaluated by the child giving the experimenter a specified number of blocks (Purpura and Lonigan, 2015), and a flashcard task was used to evaluate numeral identification. All early math assessments were administered to all three cohorts except cardinality and numeral identification, which were only administered to cohort three.

### 2.4. Analytic strategy

All factor structure analyses were conducted using M*plus* 8 (Muthén and Muthén, 2017). The analyses followed a sequence of steps to identify the factor structure model with the most support based on statistical fit indices. In total, nine *a priori* latent factor models were considered (see Supplementary Figures 1–9, which includes notes for the rationales for each model): (1) a model that included a single latent factor across constructs and timepoints, (2) a model that included a single random intercept latent factor across constructs and timepoints (all unstandardized factor loadings constrained to 1), (3) a model that included two latent factors by timepoints, one for fall and one for spring of prekindergarten, (4) a model that included two random intercept latent factors by timepoints, one for fall and one for spring of prekindergarten, (5) a model that included three latent factors by constructs (but not by timepoints), one for EF, one for mathematics, and one for language/literacy, (6) a model that included six latent factors by constructs and timepoints, one for EF in the fall and one for EF in the spring, one for mathematics in the fall and one for mathematics in the spring, and one for language/literacy in the fall and one for language/literacy in the spring, (7) a model that included a single random intercept latent factor across timepoints and constructs, and two timepoint-specific latent factors based on the remaining task-specific residual variation at each timepoint (i.e., variation independent of the random intercept), (8) one that included a single random intercept latent factor across timepoints and constructs, and three construct-specific latent factors based on the remaining task-specific residual variation for each construct (i.e., EF, mathematics, and language/literacy), and (9) one that included a single random intercept latent factor across timepoints and constructs, and six construct- and timepoint-specific latent factors based on the remaining task-specific residual variation at each timepoint and for each construct.

In all latent factor models, residual correlations among the same tasks across timepoints were included (e.g., Head-Toes-Knees-Shoulders at timepoint 1 correlated with Head-Toes-Knees-Shoulders at timepoint 2). We tested all models without this specification, but they fit the data much worse. The models without these residual correlations resulted in 13 additional degrees of freedom, but typically increased the Chi-squared test statistic by around 1,000 (indicating substantially worse fit). These alternative models without the residual correlations also did not impact the preferred model selected in our analyses. It is worth noting our model specifications are similar to other conceptualizations of bifactor models (at times are simply extensions or versions of these; e.g., Eid et al., 2016), but also include specifications of a random intercept that represents shared variance across constructs and timepoints (i.e., not just one or the other). In a simulation study, examining factor models with the random intercept were more parsimonious compared to the bifactor model, which is highly parameterized and can lead to improper solutions (Maydeu-Olivares and Coffman, 2006).

Of primary interest was which models provided the best statistical fit given the data on the 13 direct assessments of EF, mathematics, and language/literacy. Fit indices that were evaluated included the chi-squared test, Comparative Fit Index (CFI), Root Mean Square Error of Approximation (RMSEA), and Bayesian Information Criterion (BIC; Kline, 2005). In our analyses, we place emphasis on the relative fit indices of one model compared to another. Although all fit indices were evaluated for this purpose, the BIC (i.e., smaller values considered better fitting) was given priority when determining the best fitting model because not all models were nested within one another (Kline, 2005).

All models were estimated with maximum likelihood robust standard errors, which do not assume normality of variables (Muthén and Muthén, 2017). This is important as we used the raw scores on all the assessments rather than adjust for potential outliers (e.g., winsorizing). This is commonly done with these types of assessments that do not rely on reaction times. Additionally, standard errors incorporated the clustered nature of the data of children within prekindergarten classrooms with a sandwich estimator in M*Plus* 8 with the “type = complex” specification (*n* = 85). All direct assessments were centered and standardized at each timepoint (mean of 0, standard deviation of 1). This only influences the scaling of the variables, and these standardized variables are perfectly correlated with the raw scores of the variables. Standardizing variables was done so that the random intercepts (i.e., latent factor with loadings constrained equal) could reflect the equally shared variation across tasks and timepoints, rather than be impacted by task-specific scaling variance. All output and code used in this study is available from the first author by request.

#### 2.4.1. Missing data

Missing data was handled two ways: (1) all analyses used full information maximum likelihood and (2) the auxiliary variables specification for explaining missing data in M*plus* 8 was used so that socio-demographic factors could be included to help explain potential missing data patterns (gender, age at each timepoint, race/ethnicity, and treatment status). Specifically, older children at fall of prekindergarten were more likely to have missing data on all assessments, older children at spring of prekindergarten were marginally more likely to have missing data on all assessments, and children in the prekindergarten treatment status had less overall missing data on assessments. In general, very little missing data occurred that was not due to study design. Specifically, the study included three cohorts of children and three new assessments (Dimensional Change Card Sort, Cardinality, and Numeral Identification) were added for the third and final cohort, and therefore were missing for cohorts one and two. Specifically, 10 assessments were completed for all three cohorts and missing data ranged from 2.19–5.56% for timepoint 1, and 10.23%–12.87% for timepoint 2. For the three assessments only completed in cohort 3, missing data ranged from 3.97%–4.76% for timepoint 1 and was 11.11% for timepoint 2. Although cohort 3 had three additional assessments, there is no reason to expect these three variables to have fundamentally different associations with other variables by cohort (e.g., no likely explanation for why correlations between Dimensional Change Card Sort and Head-Toes-Knees-Shoulders would be fundamentally different for this cohort than the others if it was collected). Full information maximum likelihood is the recommended way to handle missing data in that it uses all available information to provide the least biased estimates (Acock, 2012).

## 3. Results

### 3.1. Descriptive statistics and correlations

Descriptive statistics, including omega reliabilities, for EF, language/literacy, and mathematics assessments are included in Table 1. Although these variables were standardized for the latent factor models (mean of 0, standard deviation of 1), we present scores on the original scale units here for direct comparison to other research using these measures. For consistency across assessments, we used the raw scores for each task. As would be expected (potentially due to both schooling and age effects), performance improved on all tasks from fall to spring of prekindergarten. Additionally, scores for all variables appeared age-appropriate and without serious floor or ceiling effects, such that variables could go one standard deviation below or above their means (with the one exception of the Head-Toes-Knees-Shoulders). Additionally, at timepoint 1, the Head-Toes-Knees-Shoulders and WJLWI both had skews greater than 1, and WJLWI at timepoint 2 had skew greater than 1, while DNS at timepoint 2 had skew less than −1.

**Table 1:** Descriptive statistics for school readiness assessments.

Task	*N*	M	SD	Min.	Max.	Skewness	Kurtosis	Reliability
**Fall**								
HTKS	661	10.9	14.8	0	59	1.18	3.17	0.96
DNS	654	19.7	8.7	0	32	−0.79	2.64	0.92
DCCS	120	10.4	5.4	2	21	0.82	2.29	0.92
PPVT	646	69.2	20.6	4	120	−0.08	2.88	0.96
WJLWI	667	7.2	4.5	0	41	1.14	7.63	--
GRTR	671	14.1	4.9	0	25	−0.11	2.42	0.80
BRLI	669	8.2	5.0	0	15	−0.10	1.54	0.93
WJAP	660	9.9	3.8	0	20	−0.23	3.21	--
PENS	667	9.2	4.9	0	22	0.31	2.54	0.90
PALM	669	10.2	3.3	0	16	−0.59	3.06	0.78
BRNC	668	8.9	6.1	0	18	−0.30	2.23	0.95
NI	120	3.9	2.7	0	9	0.21	2.06	0.86
CARD	121	2.1	1.8	0	6	0.54	2.21	0.78
**Spring**								
HTKS	596	18.1	18.3	0	60	0.56	1.94	0.97
DNS	610	22.4	7.5	0	32	−1.44	4.36	0.91
DCCS	112	13.1	6.4	3	23	0.08	1.34	0.95
PPVT	597	78.9	21.5	14	144	−0.05	2.77	0.96
WJLWI	611	9.9	5.5	0	47	1.90	11.63	--
GRTR	611	16.8	4.8	0	25	−0.56	3.02	0.83
BRLI	613	10.3	4.7	0	15	−0.80	2.24	0.93
WJAP	612	11.6	3.5	0	21	−0.11	3.50	--
PENS	604	12.3	5.3	0	24	0.02	2.35	0.90
PALM	613	11.5	2.9	0	16	−0.93	4.11	0.76
BRNC	614	11.9	5.5	0	18	−0.84	3.32	0.94
NI	112	5.3	2.7	0	9	−0.25	2.07	0.87
CARD	112	2.8	1.6	0	6	−0.01	2.31	0.69

HTKS, Head-Toes-Knees Shoulders Task; DNS, Day/Night Stroop Task; DCCS, Dimensional Change Card Sort Task; PPVT, Peabody Picture Vocabulary Test-Revised; WJLWI, Woodcock-Johnson Letter Word Identification; GRTR, Get Ready to Read; BRLI, Bracken Letter Subtest; WJAP, Woodcock-Johnson Applied Problems; PENS, Preschool Early Numeracy Scale; PALM, Math Language Assessment; BRNC, Bracken Numeracy Subtest; NI, Numeral Identification; CARD, Cardinality.

Correlations among all study variables are reported in Table 2. Of the 26 variables and 325 correlations among them, all were positive in magnitude and only four were non-significant. All four of the non-significant correlations included DNS at timepoint 1 with (1) Dimensional Change Card Sort at timepoint 1, (2) Dimensional Change Card Sort at timepoint 2, (3) Numeral Identification at timepoint 2, and (4) Cardinality at timepoint 2. Correlations typically ranged from 0.30–0.60, with the strongest correlations often between the same assessment across the timepoints.

**Table 2:** Correlations among school readiness assessments.

	1	2	3	4	5	6	7	8	9	10	11	12	13	14	15	16	17	18	19	20	21	22	23	24	25	26
**Fall**																										
1. HTKS^a^	--																									
2. DNS^a^	0.28	--																								
3. DCCS^a^	0.37	**0.17**	--																							
4. PPVT^b^	0.46	0.26	0.42	--																						
5. WJLWI^b^	0.34	0.22	0.38	0.46	--																					
6. GRTR^b^	0.45	0.30	0.50	0.62	0.68	--																				
7. BRLI^b^	0.29	0.22	0.40	0.44	0.78	0.67	--																			
8. WJAP^c^	0.49	0.29	0.43	0.60	0.49	0.65	0.48	--																		
9. PENS^c^	0.49	0.28	0.59	0.57	0.62	0.68	0.59	0.71	--																	
10. PALM^c^	0.50	0.31	0.43	0.65	0.43	0.64	0.42	0.65	0.61	--																
11. BRNC^c^	0.36	0.27	0.41	0.49	0.71	0.69	0.74	0.63	0.72	0.49	--															
12. NI^c^	0.28	0.19	0.44	0.41	0.69	0.71	0.67	0.47	0.73	0.39	0.84	--														
13. CARD^c^	0.35	0.20	0.41	0.56	0.52	0.64	0.48	0.52	0.71	0.48	0.62	0.68	--													
**Spring**																										
14. HTKS^a^	0.63	0.23	0.38	0.46	0.37	0.49	0.32	0.53	0.54	0.51	0.43	0.43	0.44	--												
15. DNS^a^	0.27	0.22	0.38	0.28	0.17	0.31	0.24	0.33	0.31	0.30	0.27	0.24	0.24	0.30	--											
16. DCCS^a^	0.29	**0.11**	0.51	0.47	0.33	0.47	0.40	0.44	0.57	0.47	0.43	0.44	0.51	0.46	0.17	--										
17. PPVT^b^	0.45	0.26	0.40	0.82	0.44	0.58	0.44	0.60	0.56	0.63	0.51	0.49	0.43	0.50	0.26	0.50	--									
18. WJLWI^b^	0.30	0.20	0.38	0.37	0.79	0.59	0.65	0.41	0.54	0.36	0.61	0.72	0.49	0.33	0.20	0.36	0.38	--								
19. GRTR^b^	0.46	0.28	0.44	0.60	0.60	0.72	0.61	0.64	0.66	0.60	0.64	0.63	0.56	0.49	0.32	0.45	0.61	0.59	--							
20. BRLI^b^	0.29	0.19	0.36	0.37	0.62	0.59	0.71	0.46	0.52	0.41	0.61	0.68	0.46	0.32	0.26	0.34	0.41	0.64	0.67	--						
21. WJAP^c^	0.49	0.25	0.46	0.56	0.49	0.60	0.46	0.71	0.65	0.61	0.59	0.52	0.47	0.52	0.33	0.51	0.59	0.45	0.64	0.46	--					
22. PENS^c^	0.49	0.26	0.50	0.58	0.60	0.67	0.58	0.68	0.73	0.57	0.68	0.67	0.65	0.52	0.31	0.54	0.61	0.55	0.70	0.55	0.68	--				
23. PALM^c^	0.46	0.19	0.35	0.61	0.36	0.54	0.36	0.59	0.56	0.72	0.42	0.35	0.39	0.52	0.31	0.43	0.63	0.32	0.58	0.41	0.61	0.57	--			
24. BRNC^c^	0.33	0.21	0.29	0.41	0.61	0.58	0.64	0.57	0.62	0.42	0.77	0.76	0.51	0.41	0.26	0.42	0.52	0.61	0.67	0.70	0.62	0.69	0.47	--		
25. NI^c^	0.17	**0.05**	0.28	0.31	0.69	0.60	0.68	0.42	0.67	0.32	0.80	0.80	0.54	0.32	0.31	0.39	0.46	0.64	0.67	0.74	0.55	0.61	0.39	0.83	--	
26. CARD^c^	0.33	0.**10**	0.32	0.45	0.43	0.55	0.43	0.50	0.59	0.49	0.53	0.53	0.52	0.49	0.34	0.30	0.48	0.38	0.59	0.47	0.48	0.65	0.47	0.56	0.55	--

Bolded values are not statistically significant at *p* < 0.05. HTKS, Head-Toes-Knees Shoulders Task; DNS, Day/Night Stroop Task; DCCS, Dimensional Change Card Sort Task; PPVT, Peabody Picture Vocabulary Test-Revised; WJLWI, Woodcock-Johnson Letter Word Identification; GRTR, Get Ready to Read; BRLI, Bracken Letter Subtest; WJAP, Woodcock-Johnson Applied Problems; PENS, Preschool Early Numeracy Scale; PALM, Math Language Assessment; BRNC, Bracken Numeracy Subtest; NI, Numeral Identification; CARD, Cardinality.

a EF Assessments.

b Literacy Assessments.

c Math Assessments.

### 3.2. Factor structure of school readiness skills

The table of fit indices for all *a priori* models (plus post-hoc models) can be found in Table 3 (Models 8 and 9 did not converge). Based on all indicators of statistical fit (i.e., χ^2^, CFI, RMSEA, BIC), model 7 was the best fitting model. Model 7 included a random intercept latent factor across timepoints and constructs, and two timepoint-specific latent factors of the remaining residual variation at the fall and spring of the prekindergarten year (χ^2^[284] = 946.04, *p* < 0.001, CFI = 0.935, RMSEA = 0.058, BIC = 38,616). Specifically, this model suggests that all variables, regardless of construct and timepoint share substantial variation (i.e., through the random intercept), and remaining task-specific residual variation largely reflected other timepoint-specific variation.

**Table 3:** Fit indices from different models estimated.

	Model fit indices					
	*(df) χ^2^*	*p*	*CFI*	*RMSEA*	*BIC*	*AIC*
Model 1: single factor	(286) 1528.39	0.00	0.878	0.080	39,162	37,768
Model 2: random intercept	(311) 1810.67	0.00	0.853	0.084	39,313	38,031
Model 3: timepoint-specific factors	(285) 1318.64	0.00	0.899	0.073	38,967	37,568
Model 4: timepoint-specific random intercepts	(309) 1617.36	0.00	0.872	0.079	39,136	37,845
Model 5: construct-specific factors	(283) 1430.98	0.00	0.887	0.077	39,088	37,680
Model 6: construct- and timepoint-specific factors	(271) 1204.92	0.00	0.908	0.071	38,947	37,484
Model 7: random intercept and timepoint-specific factors	**(284) 946.04**	0.00	**0.935**	**0.058**	**38,616**	**37,212**
Model 8: random intercept and construct-specific factors	No convergence					
Model 9: random intercept with construct- and timepoint-specific factors	No convergence					
***Post-hoc* model specifications**						
Model 7^a^: with loadings and residual correlations set to 0 if non-significant	(293) 955.11	0.00	0.935	0.057	38,566	37,203
Model 7^b^: metric invariance on the timepoint-specific factors	(301) 959.55	0.00	0.935	0.057	38,522	37,195
Model 7^c^: metric invariance on the timepoint-specific factors and residual invariance	(314) 990.01	0.00	0.934	0.056	38,485	37,217

Bolded represents the best fitting value of the 9 hypothesized models. ^a,b,c^represent *post-hoc* models based on the results of a previously hypothesized model and is not bolded for that reason.

Model 7 included some factor loadings on the timepoint-specific factors that were not significant (all factor loadings on the random intercept were significant, *p* < 0.001, and greater than 0.58). Thus, a set of post-hoc models were run. First, we removed all non-significant loadings from the timepoint-specific latent factors (i.e., set them to 0; Table 3, Model 7^a^; χ^2^[293] = 955.11, *p* < 0.001, CFI = 0.935, RMSEA = 0.057, BIC = 38,566). Then, we tested if there was metric invariance on the timepoint-specific latent factor (i.e., loadings constrained equal by task on the timepoint-space factors; Table 3, Model 7^b^; χ ^2^[301] = 959.55, *p* < 0.001, CFI = 0.935, RMSEA = 0.057, BIC = 38,522). Finally, we tested if there was residual invariance by constraining all residual variation by task to be equivalent across timepoints (Table 3, Model 7^c^; χ ^2^[314] = 990.01, *p* < 0.001, CFI = 0.934, RMSEA = 0.056, BIC = 38,485). Based on the BIC fit criteria, the preferred model had residual invariance, but based on the chi-squared test (these models were nested) only had metric invariance.

**Figure 1:**
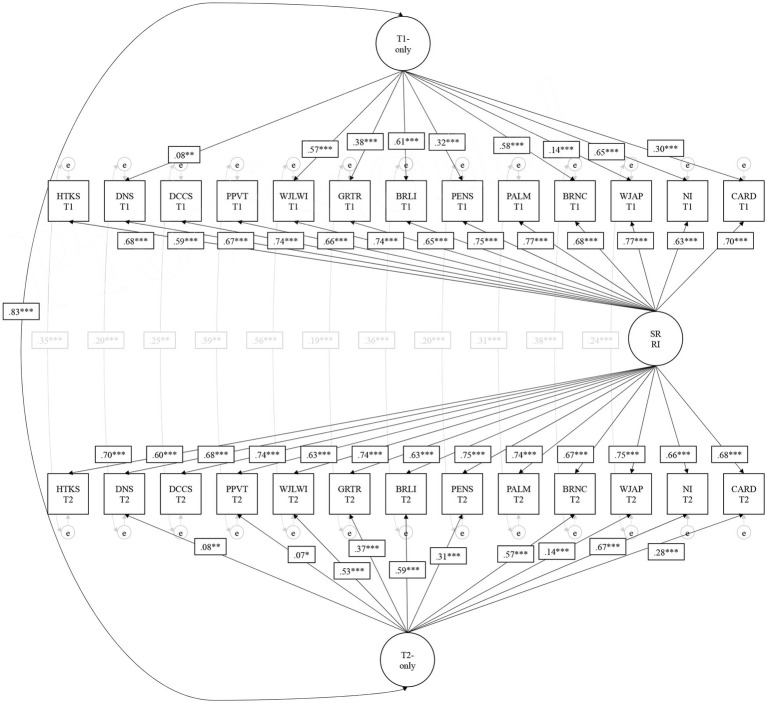
Preferred statistical model of school readiness skills in the fall and spring of Prekindergarten. Non-significant loadings have been fixed to 0 in this specification (arrows removed). Residual correlations across timepoints for NI and CARD were removed due to non-significance as well. This is Model 7^b^ in Table 3. SR RI, School Readiness Random Intercept; HTKS, Head-Toes-Knees Shoulders Task; DNS, Day/Night Stroop Task; DCCS, Dimensional Change Card Sort Task; PPVT, Peabody Picture Vocabulary Test-Revised; WJLWI, Woodcock-Johnson Letter Word Identification; GRTR, Get Ready to Read; BRLI, Bracken Letter Subtest; WJAP, Woodcock-Johnson Applied Problems; PENS, Preschool Early Numeracy Scale; PALM, Math Language Assessment; BRNC, Bracken Numeracy Subtest; NI, Numeral Identification; CARD, Cardinality. ^*^ *p* < .05. ^**^ *p* < .01. ^***^ *p* < .001.

Model 7^b^ is visually presented in Figure 1. The timepoint 1 and timepoint 2 latent factors were correlated at 0.82 (*p* < 0.001); these factors are uncorrelated with the random intercept based on the model specification (i.e., they reflect unique residual variation from what is equally shared across all tasks and timepoints). Somewhat unexpected (i.e., not hypothesized) was that Numbers/Counting, Letters, Numeral Identification, and Letter-Word Identification were the strongest loading tasks on the timepoint-specific latent factors at both timepoints (i.e., loadings greater than 0.50, *p* < 0.001). These could be representative of the skills most often targeted by prekindergarten classroom environments, and thus are more susceptible to timepoint-specific variation during the prekindergarten year, presumably due to environmental influences. The three EF tasks and receptive vocabulary were the least related to timepoint-specific variation (i.e., loadings weaker than 0.09, *p* > 0.01), suggesting they may be relatively more stable at the between-child level across the prekindergarten year (i.e., variation captured by the random intercept), the least targeted by prekindergarten environmental influences, and/or share common variation with the other school readiness tasks (i.e., less unique residual variation beyond the random intercept).

Notably, the model specifications with the random intercept and construct-specific latent factors (Model 8) and the model with a random intercept and timepoint- and construct-specific latent factors (Model 9) did not converge. However, this is likely due to the data not aligning with the models being specified. Specifically, there was more statistical support for timepoint-specific variation vs. construct-specific variation when looking at prior model specifications. That is, when comparing Model 3 (only timepoint-specific latent factors) to Model 5 (only construct-specific latent factors), Model 3 had better statistical fit suggesting that the data were more aligned with timepoint-specific variation vs. construct-specific variation.

The worst fitting model was Model 2, which only estimated a random intercept across timepoints and constructs. This is important to consider for two reasons: (1) the best fitting model was a simple extension of this with timepoint-specific latent factors specified for the residual variation across tasks at each timepoint, and (2) it underscores the tasks should not be thought of as all sharing similar variation across all timepoints (i.e., random intercept).

## 4. Discussion

The current study builds on and brings together three emerging lines of research: (1) the potential for bias in latent variable models and the need to consider alternative plausible model specifications (Bailey et al., 2018; Camerota et al., 2020; Rhemtulla et al., 2020); (2) relatively similar magnitude cross-domain correlations as within-domain correlations on these constructs in early childhood (Purpura et al., 2017a; Nguyen et al., 2019); and (3) modeling considerations for relative between-child stability in longitudinal data (Berry and Willoughby, 2017; Nesbitt et al., 2019). The most important conclusion that can be drawn from these findings is that latent variable models that do not consider the substantial role of shared variation across constructs and timepoints are likely to produce biased estimates due to factors that contribute to all constructs and relative between-child stability across timepoints. Specifically, Model 6 is most representative of how these skills are conceptualized in the field (i.e., construct- and timepoint-specific latent factors only). In Model 6, strong correlations existed among all latent factors (ranging from 0.74 to 0.95, with 6 of the 15 correlations above 0.90). If path analyses were included, one could easily infer that all timepoint 1 construct factors are strongly predictive of timepoint 2 construct factors through the structural paths. However, these estimates would be biased due to shared influences. One possible explanation is that language abilities are deeply rooted in all tasks that tap school readiness skills and it remains mostly stable at the between-child level across the prekindergarten year. Thus, it could act as factor that contributes to upwardly biased associations between EF and mathematics assessments. Additionally, including just one task to control for these potential confounding effects would likely underestimate the true effect (unless perfectly measured) and including control variables for all possible confounds is likely impossible. It is also worth mentioning this issue is potentially exacerbated when using latent variables for specific constructs because the shared variance across tasks of a construct (e.g., EF) would be subject to any factor that influences all tasks. This is not a new argument for the EF literature that has focused on task impurity concerns for specific types of EF assessments (e.g., inhibitory control tasks also capturing working memory skills), but our results suggest it may be a broader issue across early childhood school readiness assessments. In other words, when modeling shared variance across tasks of a specific construct, researchers may be capturing more of the other factors that influence performance than anticipated.

Our analyses suggested that the factor structure of including a random intercept that captures the shared variance across constructs and timepoints and includes timepoint-specific latent factors was the model that best fit the data. These findings are consistent with prior studies, as the relatively similar cross-construct correlations as within-construct correlations have been found in prior studies (e.g., Purpura et al., 2017a) including meta-analyses (Nguyen et al., 2019), and capturing relative between-child stability across development has become a central concern when estimating longitudinal models (Berry and Willoughby, 2017). One interesting and novel finding regarding the timepoint-specific latent factors was related to the tasks that loaded most strongly onto them. There was consistency across both timepoints for the four strongest loading variables: Letter-Word Identification, Letters, Numbers/Counting, and Numeral Identification. The next three strongest loading variables were: Get Ready to Read Revised, Preschool Early Numeracy Skills, and Cardinality. The weakest (or non-significant) loading variables were always: the three EF tasks, receptive vocabulary, and mathematics language. The foundational mathematical and literacy skills, followed by slightly broader mathematical and literacy skills, aligns perfectly with the skills most likely subject to the intentional targets of prekindergarten environments. EF and language abilities may be less directly targeted in prekindergarten environments, less subject to changes in between-child relational stability during this six-month period of development, more broadly involved across all tasks and timepoints (i.e., primarily captured by the random intercept), or some combination of these explanations. Changes in EF and language abilities may require more systematic and targeted efforts [e.g., interventions (Schmitt et al., 2015); curricula (Weiland et al., 2013)] than typically occurring prekindergarten environments. Further, these results do not appear to be related to measurement characteristics (i.e., skewness, kurtosis, or reliability).

### 4.1. Conceptual vs. statistical distinctions of school readiness

Although clear conceptual distinctions exist between EF, language/literacy, and mathematics tasks, there is not strong statistical evidence that the data align with these conceptual distinctions. This does not mean that models that included conceptually distinct factors always fit worse than models that did not (e.g., Model 6 vs. Model 3; Model 5 vs. Model 1). As recommended by Rhemtulla et al. (2020), it is possible that models using composite variables of common constructs may be preferred to latent variables depending on the true nature of the relations (which unfortunately is not known in non-simulation contexts). In other words, it may be that the three EF assessments all validly tap some degree of EF skills, but when latent variables are created, the shared variance among EF tasks reflects other factors that influence performance (and these other factors are similar to ones that influence mathematics and language/literacy). This is what is suggested when latent factors are not statistically distinct between EF, mathematics, and language/literacy. The data in this study were more supportive of meaningful timepoint-specific variation than construct-specific variation, with clear statistical support (i.e., by every indicator) for the model that included a random intercept with timepoint-specific latent factors. These findings are consistent with the meta-analytic results that showed mathematics tasks loaded more strongly onto latent EF than did EF tasks (Nguyen et al., 2019). Additionally, our correlation matrix did not suggest tasks within a specific construct to be more correlated with other tasks within that construct vs. other constructs, a finding seen in prior work as well (Purpura et al., 2017a). In summary, these results suggest that these 13 tasks do not show statistical support for unique construct-specific associations based on the conceptual constructs they are intended to tap.

Thus, a more fundamental issue resides with what the construct-specific latent factors represent in early childhood studies of school readiness. Do they represent general skills of the child that are present for all assessments (e.g., language, motivation, attention, persistence, engagement)? Do they reflect poor measurement of intended constructs (i.e., task impurity)? These are important questions for the field of early childhood development to further examine, across different timepoints, assessments, and samples.

### 4.2. Limitations and future directions

A number of potential limitations should be noted and suggest areas for future research. This study was fairly comprehensive in including 13 assessments of children’s EF, language/literacy, and mathematics, however, there are many measures of these skills that exist in the literature. Other tasks within these constructs, as well as within other domains of school readiness (e.g., emotional regulation), warrant further examination within this general analytic approach of considering plausible alternative models and their alignment with the data (i.e., the combination of shared and unique variation across constructs and timepoints). Relatedly, none of the models we estimated showed exceptional fit to the data (e.g., no CFIs > 0.95 and no RMSEAs < 0.05). It is possible that other model specifications exist that would better fit the data, but we encourage *a priori* approaches to testing theoretical models vs. exploratory approaches. Second the ideal length of time between assessments for capturing meaningful within-child change on these skills is unclear. It appears that such changes may have been occurring on literacy and mathematics throughout the prekindergarten year, but less so on language and EF tasks. For language and EF, are greater observational periods of change needed (e.g., 1 year), earlier developmental periods (e.g., 3 to 4 years old), or are interventions and environments that specifically target these skills needed? These are important questions for early childhood education researchers to reconcile with the known strong relation of these skills to future outcomes. All children in this sample are considered low-income and whether the observed findings would extend to other populations within this state (i.e., higher-income peers) or other states (i.e., nationally representative sample) are unknown. Finally, beyond cautions for using only a single modeling approach for drawing conclusions, it is unclear what the specific recommendation for early childhood researchers interested in modeling these school readiness skills would be based on the current study’s results. These are not simulated data where a true model of relations is known. Clearly, substantial shared variance exists across constructs and timepoints, which may influence the conclusions that can be drawn from latent model specifications that do not account for this. If researchers ignore the substantial shared variance of cross-construct associations (especially when similar in magnitude to within-construct associations), much like the relative between-child stability issue (Berry and Willoughby, 2017), estimates will likely be upwardly biased and capture other factors that contribute to all constructs being examined. It also seems likely this bias would be exacerbated when using latent variables of shared variance because what is common across tasks is likely to draw on many child and environmental factors. Researchers should challenge the use of one presumed theoretical model specification in efforts to move the field forward to a greater understanding of school readiness development across constructs and timepoints.

## 5. Conclusion

The current study examined the factor structure of cognitive aspects of school readiness (i.e., EF, language/literacy, and mathematics). The data were most consistent with a model that specified a random intercept across constructs and timepoints, with timepoint-specific latent factors (primarily composed of early literacy and mathematics skills). Consistent with recent critiques, latent variable models of school readiness skills that do not consider the substantial shared variance across constructs and timepoints are potentially miss-specified and would likely lead to biased estimates (Berry and Willoughby, 2017; Rhemtulla et al., 2020). We encourage other early childhood researchers to examine multiple theoretical model specifications over presumptions of a single theoretical model of development, with particular attention to the potential role of shared and unique variation across constructs and timepoints.

## Data availability statement

The original contributions presented in the study are included in the article/Supplementary material, further inquiries can be directed to the corresponding author.

## Ethics statement

The studies involving human participants were reviewed and approved by Purdue University IRB. Written informed consent to participate in this study was provided by the participants' legal guardian/next of kin.

## Author contributions

RD, KA, DP, and SS contributed to the conception and design of the study. JF organized the database and managed the project. RD performed the statistical analysis. RD and KA wrote the first draft of the manuscript. JF, DP, and SS reviewed and edited the manuscript. All authors contributed to manuscript and approved the submitted version.

## Funding

This study was funded by Indiana’s Family and Social Services Administration (contract # 0000000000000000000026332 and contract # F1-79-15-PK-0374) and the Purdue Research Foundation (# 60000029).

## Conflict of interest

The authors declare that the research was conducted in the absence of any commercial or financial relationships that could be construed as a potential conflict of interest.

## Publisher’s note

All claims expressed in this article are solely those of the authors and do not necessarily represent those of their affiliated organizations, or those of the publisher, the editors and the reviewers. Any product that may be evaluated in this article, or claim that may be made by its manufacturer, is not guaranteed or endorsed by the publisher.

## Author disclaimer

The content is solely the responsibility of the authors and does not necessarily represent the official views of the State of Indiana or the Indiana Family and Social Services Administration.
