# Supplementary material for: Factor structure of school readiness skills: conceptual vs. statistical distinctions
Source: Front Psychol. 2023 Jul 10;14:962651. doi: 10.3389/fpsyg.2023.962651 (PMC10363606; doi:10.3389/fpsyg.2023.962651)
Supplement: Supplementary file 1 [file Data_Sheet_1.docx]

**Supplemental Materials**

**Figure S1.** *Single Underlying Latent Factor Across Constructs and Timepoints.* This model assumes variation is shared across constructs and timepoints (some tasks loading more strongly than others), but shared variation is not construct- or timepoint-specific.

HTKS = Head-Toes-Knees Shoulders Task; DNS = Day/Night Stroop Task; DCCS = Dimensional Change Card Sort Task; PPVT = Peabody Picture Vocabulary Test-Revised; WJLWI = Woodcock-Johnson Letter Word Recognition; GRTR = Get Ready to Read; BRLI = Bracken Letter Subtest; WJAP = Woodcock-Johnson Applied Problems; PENS = Preschool Early Numeracy Scale; PALM = Math Language Assessment; BRNC = Bracken Numeracy Subtest; NI = Numeral Identification; CARD = Cardinality

**Figure S2.** *Single Underlying Latent Random Intercept Across Constructs and Timepoints.* This model is more restrictive than S1, and assumes variation is equally shared across constructs and timepoints, but is still not construct- or timepoint-specific.

HTKS = Head-Toes-Knees Shoulders Task; DNS = Day/Night Stroop Task; DCCS = Dimensional Change Card Sort Task; PPVT = Peabody Picture Vocabulary Test-Revised; WJLWI = Woodcock-Johnson Letter Word Recognition; GRTR = Get Ready to Read; BRLI = Bracken Letter Subtest; WJAP = Woodcock-Johnson Applied Problems; PENS = Preschool Early Numeracy Scale; PALM = Math Language Assessment; BRNC = Bracken Numeracy Subtest; NI = Numeral Identification; CARD = Cardinality

**Figure S3.** *Timepoint-Specific Latent Factors.* This model assumes variation is shared across constructs (some tasks loading more strongly than others) but distinct latent factors exist for timepoints (though the latent factors are correlated).

HTKS = Head-Toes-Knees Shoulders Task; DNS = Day/Night Stroop Task; DCCS = Dimensional Change Card Sort Task; PPVT = Peabody Picture Vocabulary Test-Revised; WJLWI = Woodcock-Johnson Letter Word Recognition; GRTR = Get Ready to Read; BRLI = Bracken Letter Subtest; WJAP = Woodcock-Johnson Applied Problems; PENS = Preschool Early Numeracy Scale; PALM = Math Language Assessment; BRNC = Bracken Numeracy Subtest; NI = Numeral Identification; CARD = Cardinality

**Figure S4.** *Timepoint-Specific Latent Random Intercepts.* This model is more restrictive than S3, and assumes variation is equally shared across constructs, but distinct latent random intercepts exist for timepoints (though they are correlated). HTKS = Head-Toes-Knees Shoulders Task; DNS = Day/Night Stroop Task; DCCS = Dimensional Change Card Sort Task; PPVT = Peabody Picture Vocabulary Test-Revised; WJLWI = Woodcock-Johnson Letter Word Recognition; GRTR = Get Ready to Read; BRLI = Bracken Letter Subtest; WJAP = Woodcock-Johnson Applied Problems; PENS = Preschool Early Numeracy Scale; PALM = Math Language Assessment; BRNC = Bracken Numeracy Subtest; NI = Numeral Identification; CARD = Cardinality

**Figure S5.** *Construct-Specific Latent Factors.* This model assumes variation is shared across timepoints (some tasks loading more strongly than others) but distinct latent factors exist for constructs (though the latent factors are correlated). HTKS = Head-Toes-Knees Shoulders Task; DNS = Day/Night Stroop Task; DCCS = Dimensional Change Card Sort Task; PPVT = Peabody Picture Vocabulary Test-Revised; WJLWI = Woodcock-Johnson Letter Word Recognition; GRTR = Get Ready to Read; BRLI = Bracken Letter Subtest; WJAP = Woodcock-Johnson Applied Problems; PENS = Preschool Early Numeracy Scale; PALM = Math Language Assessment; BRNC = Bracken Numeracy Subtest; NI = Numeral Identification; CARD = Cardinality

**Figure S6.** *Construct- and Timepoint-Specific Latent Factors.* This model assumes variation is distinct by construct and timepoint (though the latent factors are all correlated). HTKS = Head-Toes-Knees Shoulders Task; DNS = Day/Night Stroop Task; DCCS = Dimensional Change Card Sort Task; PPVT = Peabody Picture Vocabulary Test-Revised; WJLWI = Woodcock-Johnson Letter Word Recognition; GRTR = Get Ready to Read; BRLI = Bracken Letter Subtest; WJAP = Woodcock-Johnson Applied Problems; PENS = Preschool Early Numeracy Scale; PALM = Math Language Assessment; BRNC = Bracken Numeracy Subtest; NI = Numeral Identification; CARD = Cardinality

**Figure S7.** *Single Underlying Latent Random Intercept and Timepoint-Specific Latent Factors.* This model is an extension of S2 and S3, assuming a single underlying random intercept (shared variation across constructs and timepoints), with remaining residual variation contributing to timepoint-specific latent factors only (that are correlated). HTKS = Head-Toes-Knees Shoulders Task; DNS = Day/Night Stroop Task; DCCS = Dimensional Change Card Sort Task; PPVT = Peabody Picture Vocabulary Test-Revised; WJLWI = Woodcock-Johnson Letter Word Recognition; GRTR = Get Ready to Read; BRLI = Bracken Letter Subtest; WJAP = Woodcock-Johnson Applied Problems; PENS = Preschool Early Numeracy Scale; PALM = Math Language Assessment; BRNC = Bracken Numeracy Subtest; NI = Numeral Identification; CARD = Cardinality

**Figure S8.** *Single Underlying Latent Random Intercept and Construct-Specific Latent Factors.* This model is an extension of S2 and S5, assuming a single underlying random intercept (shared variation across constructs and timepoints), with remaining residual variation contributing to construct-specific latent factors only (that are correlated). HTKS = Head-Toes-Knees Shoulders Task; DNS = Day/Night Stroop Task; DCCS = Dimensional Change Card Sort Task; PPVT = Peabody Picture Vocabulary Test-Revised; WJLWI = Woodcock-Johnson Letter Word Recognition; GRTR = Get Ready to Read; BRLI = Bracken Letter Subtest; WJAP = Woodcock-Johnson Applied Problems; PENS = Preschool Early Numeracy Scale; PALM = Math Language Assessment; BRNC = Bracken Numeracy Subtest; NI = Numeral Identification; CARD = Cardinality

**Figure S9.** *Single Underlying Latent Random Intercept and Construct- and Timepoint-Specific Latent Factors.* This model is an extension of S2 and S6, assuming a single underlying random intercept (shared variation across constructs and timepoints), with remaining residual variation contributing to construct- and timepoint-specific latent factors (that are correlated). HTKS = Head-Toes-Knees Shoulders Task; DNS = Day/Night Stroop Task; DCCS = Dimensional Change Card Sort Task; PPVT = Peabody Picture Vocabulary Test-Revised; WJLWI = Woodcock-Johnson Letter Word Recognition; GRTR = Get Ready to Read; BRLI = Bracken Letter Subtest; WJAP = Woodcock-Johnson Applied Problems; PENS = Preschool Early Numeracy Scale; PALM = Math Language Assessment; BRNC = Bracken Numeracy Subtest; NI = Numeral Identification; CARD = Cardinality

**Supplemental Table 1.** *Additional Measurement Information*

| Executive Function Tasks | | |
| --- | --- | --- |
| Measure | Procedures | |
| Head-Toes-Knees-Shoulders (HTKS; McClelland et al., 2014) | Skills Assessed: Working memory, inhibitory control, and cognitive flexibility.  Task: Children were taught four rules that were presented as pairs (e.g., “touch your head/toes,” “touch your knees/shoulders”). Children were told to do the *opposite* of what the research assistant said (e.g., touch their toes when the research assistant said, “touch your head”). The testing component included three sections of 10 items (i.e., 30 items total) which become more difficult as children progressed through the task and were required to remember new rules (e.g., touch their shoulders when the research assistant said, “touch your toes”).  Scoring: Children were awarded 2 points for a correct response, 1 point for self-correcting an incorrect response, and 0 points for an incorrect response. To advance to the next section, children had to score at least 4 points on the preceding section.  Psychometric Properties: In our sample, the HTKS demonstrated excellent internal consistency (ω_fall_ = .96; ω_spring_ = .97). | |
| Day/Night Stroop (DNS; Gerstadt et al., 1994) | Skills Assessed: Inhibitory control.  Task: Children were shown a set of 16 cards that each had a drawing of a sun (“day”) or a moon (“night”) on them. During this task, children were shown one card at a time and had to say the *opposite* time of day that was represented by the card (i.e., say “night” when shown a picture of the sun; say “day” when shown a picture of the moon).  Scoring: Children were awarded 2 points for every correct answer, 1 point for answers that were self-corrected or similar to the correct answer (e.g., saying “sun” instead of “day”), and 0 points for incorrect responses.  Psychometric Properties: The DNS has demonstrated strong internal consistency when used with preschoolers (e.g., Gerstadt et al., 1994; McClelland et al., 2014; Schmitt, Purpura, et al., 2019), including in the current sample (ω_fall_ = .92; ω_spring_ = .91). | |
| Dimensional Change Card Sort* (DCCS; Zelazo, 2006) | Skills Assessed: Cognitive flexibility.  Task: Children were presented with four boxes with unique pictures (a big blue dog, a small yellow bird, a medium-sized red fish, and a frog [a “distraction’ box]) attached to them and were asked to place cards into the box that corresponds with the research assistant’s instructions. The task included four phases that require children to sort 6 cards based on specific dimensions. First, children were asked to sort cards based on their shape (e.g., a card with a dog on it would be placed in the box with the picture of the dog, regardless of its size and color). Second, children had to arrange cards according to their color (e.g., a card with a red dog would be placed in the box with a red animal on it, regardless of the shape or size of the picture on the box). Third, children arranged the cards into corresponding boxes based on size (e.g., a card with a large animal on it would be placed in the box with a large animal, regardless of the shape or color of the picture on the card). If children correctly sorted at least 5 of the 6 cards during the size phase, they moved on to the fourth phase of the task which required children to sort according to previous dimensions based on the presence or absence of a border on the card.  Scoring: Children were awarded 1 point for each correct answer, for a maximum possible score of 24 points. Although different scoring methods exists for card sorting tasks, we followed the same protocol used in a prior study using this specific assessment (McClelland et al., 2014).  Psychometric Properties: This iteration of the DCCS has been used in previous studies (e.g., Schmitt, Korucu, et al., 2019) and has demonstrated strong reliability in previous studies (McClelland et al., 2014) as well as the current sample (ω_fall_ = .92; ω_spring_ = .95). | |
| Language/Literacy Tasks | | |
| Measure | | Procedures |
| Peabody Picture Vocabulary Test (PPVT; Dunn & Dunn, 2007) | | Skills Assessed: Receptive vocabulary.  Task: The PPVT is administered using a stimulus book that includes four illustrations for each testing item. For each item, the research assistant asked the child to point to the picture that matches the word they said (e.g., “Put your finger on the picture that shows *laughing).*  Scoring: The PPVT consists of 19 age-based sets that includes 12 items each, for a total of 228 items. For brevity, children’s starting point for the assessment is determined based on their age. If children did not correctly identify at least 11 items within their starting set, the assessment is continued using the previous item set until a basal level has been established. Children earned one point for each picture they correctly identified. The assessment continued until children responded incorrectly to 8 or more items within the same set.  Psychometric Properties: The PPVT is a common assessment of vocabulary skills for both children and adults and has strong psychometric properties. In our sample, the PPVT demonstrated strong internal consistency (ω_fall_ = .96; ω_spring_ = .96), corroborating the results of past studies (Dunn & Dunn, 2007). |
| Letter-Word Identification subtest of the Woodcock-Johnson (WJLWI; Schrank et al., 2014) | | Skills Assessed: Letter and word recognition, decoding skills.  Task: Children were presented with a list of letters and/or words and were asked to verbally identify them (e.g., “What letter is this?) or point to the letter or word that the assessor asked them to identify.  Scoring: The WJLWI includes 76 items grouped into 15 sets. Children earned one point per correct answer, for a possible high raw score of 76. The test administrator suspended testing after the child incorrectly responded to six items in a row at the end of a set.  Psychometric Properties: Reviews have demonstrated that subtests of the Woodcock-Johnson Tests of Achievement demonstrate acceptable to excellent reliability (Villarreal, 2015). |
| Letters subtest of the Bracken Basic Concept Scale (BRLI; Bracken, 2006) | | Skills Assessed: Letter recognition.  Task: During the BRLI, children were shown pages of the stimulus book that have several letters (uppercase and/or lowercase) scattered across them. They were asked to identify specific letters by pointing to the letter on the page that corresponded with the research assistant’s instructions (e.g., Show me, “A”).  Scoring: The BRLI consists of 15 items and children earned 1 point for each correct answer. If a child incorrectly identified 3 letters in a row, the research assistant discontinued the subtest.  Psychometric Properties: Past research suggests that the Bracken Basic Concept Scale (which includes the BRLI and other subtests such as colors and shapes) is a reliable and valid measure of basic academic skills among preschoolers (Bracken, 2006), which is reflected by our sample (ω_fall_ = .93; ω_spring_ = .93). |
| Get Ready to Read Revised (GRTR; Lonigan & Wilson, 2008) | | Skills Assessed: Emerging reading skills.  Task: Children were shown four pictures from a stimulus book and asked to point to the picture that demonstrated the answer the research assistant’s question (e.g., “These are pictures of a cereal box. Which picture tells you the name of the cereal?”).  Scoring: Children were administered all 25 items of the GRTR and were awarded 1 point for each correct response (maximum score = 25).  Psychometric Properties: Previous studies demonstrate that the GRTR has adequate reliability and strong predictive validity (Phillips et al., 2009; Whitehurst, 2001); the GRTR also exhibited good reliability in the current sample (ω_fall_ = .80; ω_spring_ = .83). |
| Early Mathematics Skills | | |
| Measure | Procedures | |
| Applied Problems subtest of the Woodcock-Johnson (WJAP; Schrank et al., 2014) | Skills Assessed: Basic early mathematics skills (e.g., counting, addition, subtraction).  Task: The WJAP includes 63 problems grouped into 14 sets that assess early mathematics skills through word or story problems (Schrank et al., 2014). Each item was read aloud to the child by the administrator and was accompanied with an illustration in the stimulus book (e.g., “how many dogs are there in this picture?”). Children proceeded through the assessment until they incorrectly responded to five items at the end of a set.  Scoring: Children were awarded 1 point for correct answers and 0 points for incorrect answers or abstentions, for a score range of 0-63.  Psychometric Properties: Previous reviews have suggested that the subtests of the Woodcock Johnson Tests of Achievement exhibit acceptable to excellent reliability (Villarreal, 2015). | |
| Preschool Early Numeracy Skills (PENS; Purpura et al., 2015; Purpura & Lonigan, 2015) | Skills Assessed: Basic early mathematics skills (e.g., counting, addition, subtraction).  Task: The PENS measures children’s early numeracy skills is administered using a stimulus book that includes illustrations that children interact with to answer items (e.g., counting a set of 11 dots).  Scoring: Children received 1 point for each correct response (score range 0-24) and the assessment continued until the child incorrectly responded to three consecutive items  Psychometric Properties: The PENS has demonstrated high internal consistency (α = .93) in previous studies (Purpura & Reid, 2016) and in the current sample (ω_fall_ = .90; ω_spring_ = .90). | |
| Numbers/Counting subtest of the Bracken Basic Concept Scale (BRNC; Bracken, 2006) | Skills Assessed: Basic early mathematics skills (e.g., counting, addition, subtraction).  Task: For the first set of items of the subtest, children were shown pages of the stimulus book that had several numerals printed across the pages. For each of the items, the child was asked to identify a specific numeral (e.g., Show me the “two”). For the remaining items, children were shown a page in the stimulus book with four pictures that represented a different number of items. Children were asked to point to the picture that had *n* items (e.g., “point to nine ducks”).  Scoring: Children were awarded 1 point for each correct answer for a maximum possible score of 18. If children incorrectly answered 3 items in a row, the assessment was discontinued.  Psychometric Properties: Previous research studying the use of the Bracken Basic Concept Scale for prekindergarten-age children has suggested that it is a valid and reliable measure of basic academic skills (Bracken, 2006). The BRNC also demonstrated strong internal consistency in the current study (ω_fall_ = .95; ω_spring_ = .94). | |
| Preschool Assessment of the Language of Mathematics (PALM; Purpura & Logan, 2015; Purpura & Reid, 2016) | Skills Assessed: Quantitative and spatial language skills.  Task: The PALM is administered using a stimulus book that includes illustrations that children interacted with to answer items (e.g., “point to the dot that is *under* the boy” or “which sets have the *same* number of dots?”).  Scoring: Children were administered all the items of the assessment and were awarded 1 point for each correct response (score range of 0-16).  Psychometric Properties: Previous studies have demonstrated the PALM to have good internal consistency (α = .85; Purpura & Logan, 2015), though the internal consistency of the PALM was slightly weaker in the current sample (ω_fall_ = .78; ω_spring_ = .76). | |
| Cardinality* (CARD; Purpura & Lonigan, 2015) | Skills Assessed: Cardinality (i.e., the understanding that each number represents a discrete quantity).  Task: Children were provided with a set of 10 blocks and were asked to give the research assistant a smaller set of blocks (e.g., “give me 4 blocks”) for three specific quantities. Then, they were provided with an additional 10 blocks (for a total of 20 blocks) and were asked to give the research assistant a smaller set of blocks (e.g., “give me 16 blocks”) for three specific quantities.  Scoring: If the child gave the research assistant the correct number of blocks, they earned one point, with possible scores ranging from 0-6.  Psychometric Properties: Though the internal consistency of the CARD was acceptable at the fall timepoint (ω_fall_ = .78), it was slightly weaker in the spring (ω_spring_ = .69). | |
| Numeral Identification* (NI; Purpura & Lonigan, 2015) | Skills Assessed: Number identification  Task: Each child was shown a set of nine flashcards that had one single- or double- digit number (i.e., 1, 2, 3, 7, 8, 10, 12, 14, 15) printed on them. Children were shown the flashcards one at a time in a random shuffled order and were asked to say the name of the number presented on the card  Scoring: Children received 1 point for each correct answer for a maximum possible score of 9.  Psychometric Properties: Previous studies have demonstrated that the NI task has strong internal consistency (α = .90; Purpura & Lonigan, 2015), though this was slightly weaker in the current sample (ω_fall_ = .86; ω_spring_ = .87). | |

*Administered for Cohort 3 only.
